# Supplementary material for: Polyimide Modified with Different Types and Contents of Polar/Nonpolar Groups: Synthesis, Structure, and Dielectric Properties
Source: Polymers (Basel). 2025 Mar 13;17(6):753. doi: 10.3390/polym17060753 (PMC11944749; doi:10.3390/polym17060753)
Supplement: Supplementary file 1 [file polymers-17-00753-s001.zip › polymers-3454476-supplementary.pdf]

Polymide Modified with Different Types and Contents of Polar/No-polar Groups: Synthesis, Structure and Dielectric Properties

Ting Li [1,2,*], Jie Liu [2], Shuhui Yu [2], Xiaojun Zhang [1,*] and Zhiqiang Chen [1]

[1] Arrayed Materials (China) Co., Ltd., Shenzhen 518131, China
[2] Shenzhen Institute of Advanced Technology, Chinese Academy of Sciences, Shenzhen 518055, China
* Correspondence: ting.li@arrayedmaterials.com (T.L.); xiaojun.zhang@arrayedmaterials.com (X.Z.)

2.1 Materials

Bis(4-(3-amino phenoxy)phenyl)methanone (BABP 99%) was purchased from Shanghai Bide Pharmaceutical Technology Co., Ltd. Benzophenone-3,3,4',4'-tetracarboxylic dianhydride (BPDA 98%) was purchased from Sahn Chemical Technology (Shanghai) Co., Ltd. 2,2'-Bis(trifluoromethyl)benzidine (99%) was purchased from Shanghai Aladdin Reagent Co., Ltd. Commercially available acetone, anhydrous ethanol, N, N-dimethylacetamide (DMAc), Chromatographic grade N, N-dimethylformamide (DMF), dimethyl silicone oil and all reagents were used as received.

2.2 Synthesis and preparation of polyimides

To eliminate experimental confounding factors, we chose to fix the molar ratio of diamine and dianhydride monomers at 1:1. The specific copolymerization ratio is shown in Fig.1 and table.1. Taking the preparation of polyimide film with the code 2B/3T/B as an example, the process is as follows (Fig.S1):

Under a nitrogen atmosphere, 1.4976 g of bis(4-(3-aminophenoxy)phenyl)methane (BABP) and 0.7252 g of 2,2′-bis(trifluoromethyl)-4,4′-diaminobiphenyl (TFMB) were added to a three-neck flask. Then, 16.67 mL of N, N-dimethylacetamide (DMAc) was added to the reaction vessel. The mixture was stirred at a low speed in an ice bath until the monomers were completely dissolved. Within half an hour, a total of 1.9334 g of dianhydride 3,3′,4,4′-benzophenone tetracarboxylic dianhydride (BPDA) was added in small portions multiple times. After the feeding was completed, the ice bath apparatus was removed. The reaction was then stirred for 22 hours to obtain a polyimide acid. The glass substrate was treated with acetone/alcohol/deionized water separately by ultrasonic treatment for 15 minutes. The glass surface was completely dried with a lint-free cloth. The above-mentioned polyimide acid was spin-coated onto the glass surface using a spin coater (WS-650Mz-23NPPB). The coated glass was transferred to a vacuum oven and dried at 65 °C for 6 hours. Then, it was transferred to a

tube furnace under an inert atmosphere. The thermal imidization process was carried out with the following program: curing at 120°C for 1 hour, 180°C for 1 hour, 240°C for 2 hours, 280°C for 2 hours, and 320°C for 1 hour. After that, the film was soaked in a water solution for 4 hours, peeled off from the substrate surface, and placed in a vacuum oven at 120°C for 2 hours drying.

Table S1. Polyimides with different copolymerization ratios and sample Description

**Material**		**The proportion of PAA ingredients**						
**Diamine**	BABP	0	0.2	0.4	0.5	0.6	0.8	1
	TFMB	1	0.8	0.6	0.5	0.4	0.2	0
**Dianhydride**	BPDA	1	1	1	1	1	1	1
**Sample Description**		PI-100%	PI-80%	PI-60%	PI-50%	PI-40%	PI-20%	PI-0%

During the preparation of PI via polycondensation, the most critical step is determining the stoichiometric ratio of diamine to dianhydride. This is because PI are formed through the condensation reaction of these two monomers, and an improper ratio can lead to the formation of by-products, which can adversely affect the final properties of the PI. Typically, to obtain the prepolymer (polyamic acid, PAA), the ideal stoichiometric ratio is 1:1, meaning that each diamine molecule corresponds to one dianhydride molecule. At this ratio, the two monomers can react more completely, forming a stable polymer chain.

In our study, we used two different diamine monomers and one dianhydride monomer for copolymerization, still maintaining stoichiometric ratio of diamine: dianhydride is 1:1. Therefore, we ensured that the total amount of BABP and TFMB (diamine monomers) was equal to the amount of BPDA (dianhydride monomer). We varied the ratio of TFMB to the total diamine monomers in 20% increments and named the copolymers based on the proportion of TFMB in the total diamine monomers.

Table S2. GPC data for PI with different copolymerization ratios and sample Description

**Polymer**	**PI-100%**	**PI-80%**	**PI-60%**	**PI-50%**	**PI-40%**	**PI-20%**	**PI-0%**
**Mn**	42669	46510	45125	45656	43530	47233	41414
**Mw**	50715	58510	54839	55289	52088	58717	48727
**PDI**	1.1886	1.2484	1.2153	1.211	1.1966	1.2431	1.1766

Mn: number-averaged molecular weight; Mw: weight-average molecular weight; Polydispersity (PDI): The ratio of the weight average molecular weight to the number average molecular weight is called the polydispersion coefficient (PDI=Mw/Mn).

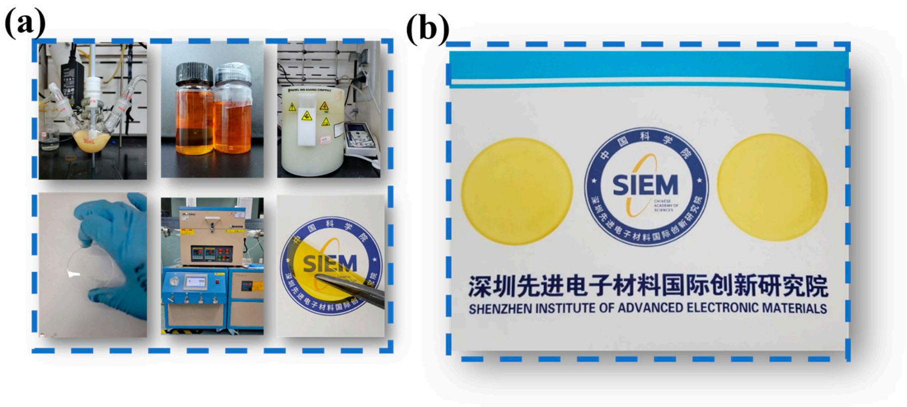

Figure S1. (a) Physical photos during the preparation of the PI films; (b) A photograph of the PI-100% PI film.

2.3 Characterization

Before testing, the film sample was cut into a rectangle of 30*3 mm, the stress-strain curve of films were Measured by TA DMA Q800 under a strain rate of 10 mm/min. Thermalgravimetric analysis (TGA) was performed in N_2 atmosphere with a heating rate of 10 °C/min and a temperature range of 25°C to 800°C. The differential scanning calorimeterr (DSC) (TA Q2000@Mfg-dsc) was used in N_2 atmosphere from 25°C to 300°C at a rate of 5 °C/min. Au electrodes with diameters of 4mm were sputtered on both sides of the PI film by a magnetron sputtering instrument, and the dielectric properties were tested with 4294A analyzer and EC1a oven (PK-CPT1705, PolyK Technology Co., Ltd., China), the frequency range is 10^2-10^6Hz.

Using the ENA series network analyzer (E5071C 300 KHz-20 GHz) to test the high-frequency dielectric properties. The PI film sputtering with Au electrode was immersed in dimethyl silicone oil, and the breakdown strength was tested with a dielectric strength tester (CS9912BX, ANO Instrument Technology Co., Ltd., China). Each sample was tested at least 30 points. The data points were arranged in numerical order, and the middle 15 values were selected. The Weibull distribution is used to assess the reliability of breakdown strength, and it is expressed in Formula 1. In the formula, E_b represents the cumulative breakdown probability, E represents the electric field strength, α is the scale factor that represents the breakdown strength when the cumulative breakdown probability is 63.2%, also known as characteristic breakdown strength. β is the shape factor that describes the distribution of breakdown strength. Generally, a larger β value indicates a narrower distribution of breakdown strength and better reliability of the material. [1-2]

$$E_b = 1 - \exp\left[-\left(\frac{E}{\alpha}\right)^{\beta}\right] \quad \text{(S1)}$$

The testing conditions for the energy storage performance of the film are similar to those for breakdown strength testing. The piezoelectric polarization instrument was used to measure the polarization-electric field hysteresis loop of the film samples at 25°C and 150°C. Additionally, the charge-discharge energy density and efficiency were evaluated. The molecular weight of polymer molecules was performed by gel permeation chromatography (GPC) with Chromatographic grade N, N-dimethylformamide（DFM） as the mobile phase to test.

The UV-Vis-NIR diffuse reflectance spectroscopy (UV-Vis-NIR) was employed to characterize the samples using ultraviolet visible spectrophotometer, the scanning range was from 200 to 4000 nm. In the absorption spectrum curve, a tangent line is drawn at the point of maximum change along the curve, and the intersection of the tangent line with the x-axis is recorded as λ_g. The bandgap width (E_g) is then calculated by utilizing the following equation 2, the bandgap (E_g) width of the samples can be calculated[3]. Fourier transform infrared spectrum (FTIR) analysis tested by Fourier-transform infrared spectroscop, the wavelength range is 4000-400mm. The X-ray diffraction (XRD) patterns from 5° to 80° were recorded by Rigaku-Dmax 2500 diffractometer under Cu K α radiation.

$$E_g = \frac{1240}{\lambda_g} \quad \text{(S2)}$$

In our work, the stress-strain tests were conducted in strict accordance with established standards: the testing methodology, experimental conditions, and specimen dimensions were meticulously designed and implemented following the specifications outlined in references ASTM Standards documents: D3039/D3039M − 17 “Standard Test Method for Tensile Properties of Polymer Matrix Composite Materials” and GB1040-79 “Plastics-Determination of tensile properties”.

The experimental procedure was meticulously conducted according to the following protocol: 1) The PI films were precisely fabricated into dumbbell shapes using laser cutting technology, the dimensional parameters of each sample are detailed in table S3, The film thickness at the central region of the dumbbell-shaped specimens was measured and recorded. 5 specimens were prepared for each test condition to ensure statistical reliability. 2) The samples were securely clamped in the tensile testing fixtures. 3) Parameter Setting: critical testing parameters were configured, including: loading rate, thickness of films, environmental conditions. 4) Testing Execution: following system verification, the test was initiated. The crosshead moved at a constant velocity while the data acquisition system simultaneously

recorded the load-elongation curve, And the test continued until specimen fracture occurred. 5) Repetition and Validation: the procedure was repeated for the remaining four specimens, data uniformity was maintained within ±5%. Outliers were identified and excluded based on statistical analysis.

Table S3. Tensile Specimen Geometry Requirements

Designation	Total length	X of the middle parallel part		The end width of dumbbell	The photo of testing sample
		Length	width		
Size /mm	110	9.5±2.0	25±0.4	45	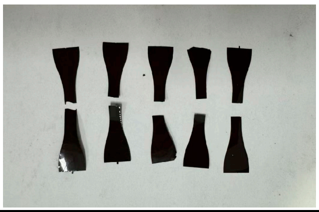
Parameter	L	C	b	W	

2.4 DFT calculation methods

Electrostatic-derived HOMO and LUMO Functions and Their Vicinity

Using the $DMol_3$ computational module, we conducted calculations to determine the energy levels and the functions surrounding the Highest Occupied Molecular Orbital (HOMO) and the Lowest Unoccupied Molecular Orbital (LUMO). The chosen computational parameters included the Generalized Gradient Approximation (GGA) functional framework and the BLYP exchange-correlation functional method[4-7].

Thermodynamic Simulation

To construct an amorphous mixed cell, we employed the Amorphous Cell module. The preliminary step involved performing geometry optimization using the Forcite Calculation module. Within the Geometry Optimization setting under the Energy option, the COMPASS II force field was selected to optimize the geometric structure. Subsequently, an annealing process was conducted at temperatures ranging from [insert temperature range, e.g., 150°C to 250°C], similar to the experimental polymerization temperatures. This was achieved by first performing short-duration molecular dynamics simulations under constant temperature conditions using the NVT ensemble in the Ensemble setting. Following this, simulations were continued under constant energy conditions using the NVE ensemble. Finally, the cohesion energy of the post-simulation configurations was calculated to evaluate the thermodynamic properties of the system.

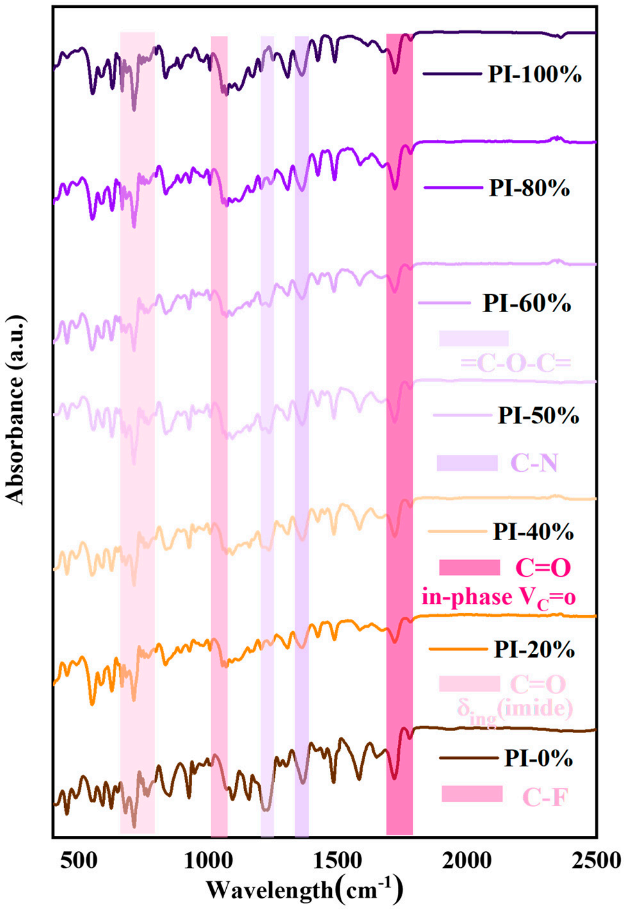

Figure S2. The FTIR of polyimide films with different copolymer ratios.

In the Fig S3, it can be determined that the characteristic peak positions of the absorption band representing the carboxyl group in PAA, such as 1659 cm^{-1} and 1718 cm^{-1}, completely disappear. This indicates that the amide groups in PAA completely disappear after cyclization with the carboxyl groups to form polyimide. [8]

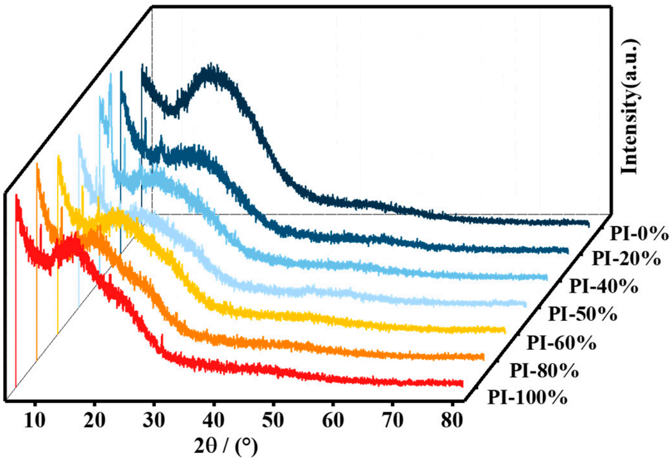

Figure S3. The XRD of polyimide films with different copolymer ratios.

XRD (X-ray diffraction) testing of the PI (polyimide) film exhibits a broad amorphous morphology with scattering peaks at $2\theta \approx 10^{\circ}$-$20^{\circ}$.[9] This scattering peak is attributed to the π-π stacking between the imide rings and benzene rings. According to the Bragg equation, the d-spacing trend from PI-0% to PI-100% is as follows： 5.35, 4.54, 5.21, 4.7, 4.21, 5.02, 4.87 and 5.42, which means that a more stable configuration forms with the increased PI contains. A larger value of d means that the molecular density is lower, the chain arrangement becomes looser, and the chain spacing is greater. With the increase of TFMB content, d first decreased and then increased, indicating that the structural change of diamine monomer had a significant effect on the chain conformation. Copolymerization made the internal crystal plane spacing of PI smaller and the density increased.

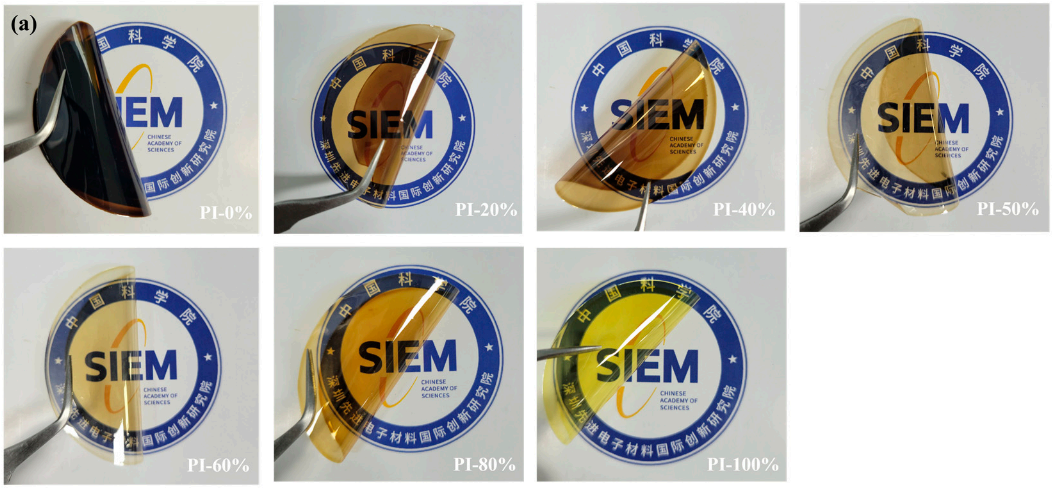

Figure S4. Optical photographs of PI films with different copolymerization ratios

The color of the PI films varies systematically with different co-polymer proportions. As the TFMB content increases, the color of the PI films gradually becomes lighter. One reason for this color change is that the large-volume side groups in TFMB weaken the charge transfer complexes (CTC) in the polymer molecular chains. Another reason is the variation in carbon content. The color of PI-0% is the darkest because the BABP monomer contains more carbon atoms and has a longer chain length compared to TFMB, making it easier to form long conjugated structures that absorb more visible light, resulting in a darker color and higher carbon content. Additionally, the molecular structure changes during the thermal treatment process also affect the final color of the PI films.

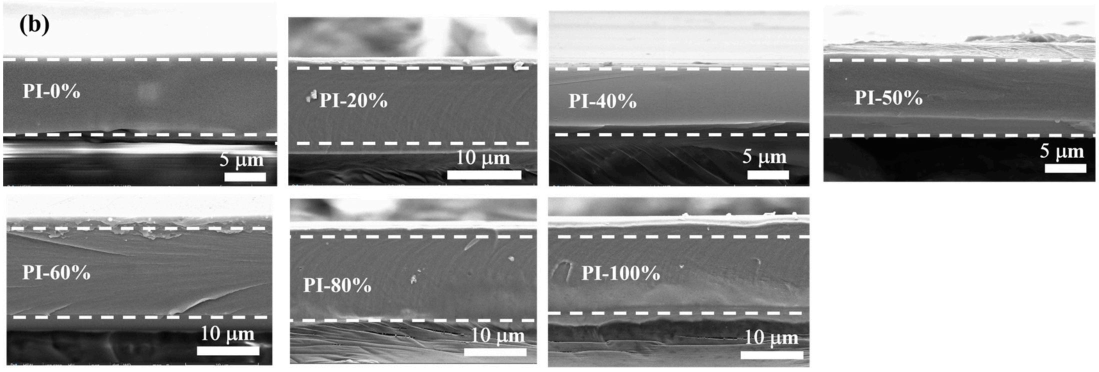

Figure S5. The Section morphology of polyimide films with different copolymer ratios at 25°C.

The PI film is cut into rectangle (3*0.5 cm), after marking, it is immersed in liquid nitrogen for 10 minutes. It is then quickly removed and gently broken using flat tweezers. Next, it is fixed on a section holder using conductive adhesive and coated with gold for 30 seconds. The morphology is observed under SEM. It can be observed that the entire series of PI films exhibit good density, with almost no presence of voids or cracks. This provides a solid foundation for achieving high mechanical and electrical insulation properties.

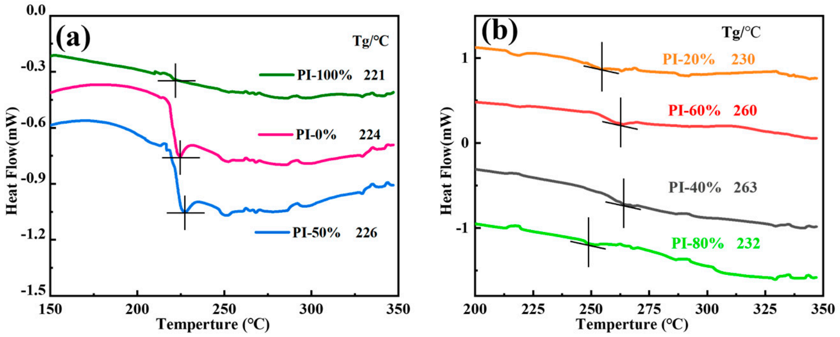

Figure S6. The DSC of polyimide films with different copolymer ratios.

After compounding TFMB and BABP, the glass transition temperature (T_g) increased, as shown in Fig S2, the T_g of the PI-40% film reaches the highest value at 263°C, whereas the non-copolymerized PI film has a T_g of approximately 220°C. The elevation of T_g can be attributed to the increased chain packing density under the copolymerization ratio of PI-60% and PI-40%, which hinders molecular chain mobility.

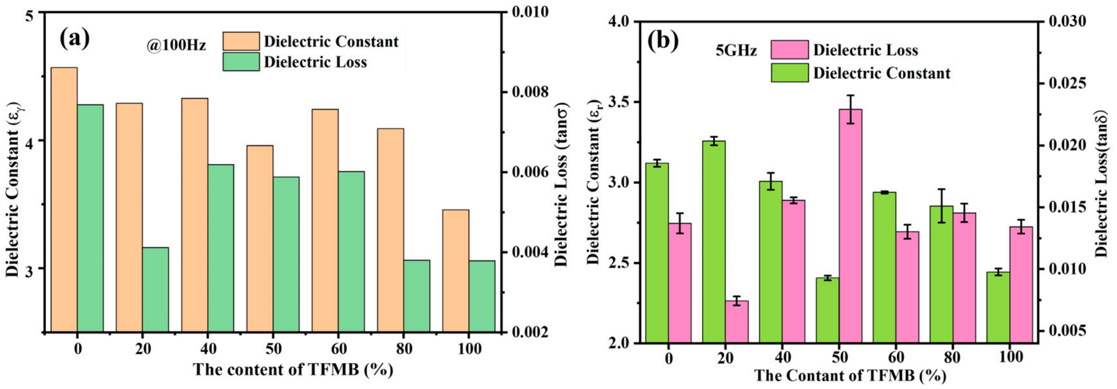

Figure S7. The high frequency (5GHz) dielectric constant and dielectric loss of polyimide films with different copolymer ratios at 25°C.

The chromatographic column of GPC is to arrange and separate the molecules in the order from large to small. The passage time of large molecular weight is short, and the passage time of small molecular weight is long. The outflow curve represents this process. The copolymerization of the two monomers can effectively increase the molecular weight of PI, which verifies the conjecture that the crosslinking density of the polymer molecular chain increases.

Table S4. Summary of thermal and mechanical properties of PI films with different copolymerization ratios

Polymer	Tensile Strength (Mpa)	Tensile Modulus (Gpa)	Elongation at Break (%)	T_g (°C)	$T_{w,5\%}$	Char Yied (%)
PI-0%	87±11	0.9±0.1	10±1	217	447	72.3
PI-20%	102±4	1±0.5	10±3	224	448	62.3
PI-40%	105±7	1±0.4	11±3	234	436	55.6
PI-50%	98±5	1.2±0.6	8±2	236	403	53.8
PI-60%	120±11	1.3±0.9	9±3	253	451	56.5
PI-80%	99±16	1.4±0.2	7±0	240	485	68.8
PI-100%	113±2	1.6±0.6	7±3	219	489	50.3

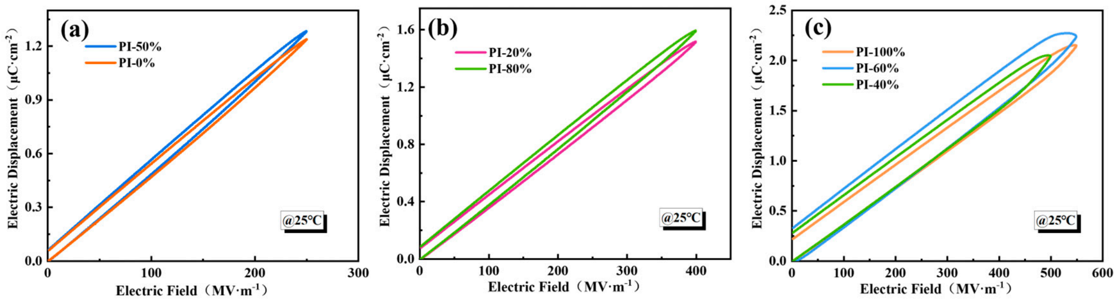

Figure S8. (a-c) The D-E loop of polyimide films with different copolymer ratios at 25°C.

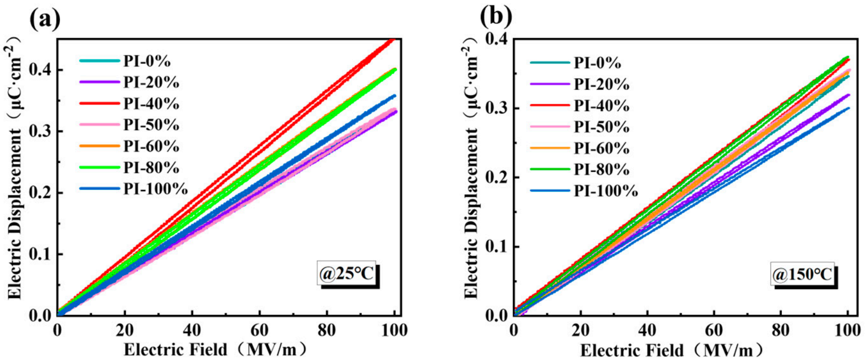

Figure S9. The D-E loop of polyimide films with different copolymer ratios at (a) 25 °C and (b)150 °C under 100 MV/m.

According to the classical electromagnetic theory, the electric displacement (D) of the material caused by the electric field intensity of E is EdD, for linear dielectrics, the dielectric permittivity is independent on the electric field strength, ε_0 is the vacuum dielectric permittivity, and the U_e can be expressed using the following equation 3: [10-11]

$$U_e = \int_0^{D_{max}} E\,dD = \int_0^{E_{max}} E\,dD = \frac{1}{2}\varepsilon_0\varepsilon_r E^2 \qquad \text{(S3)}$$

2.5 DFT calculations.

Perform calculations on energy levels and nearby functions using the DMol3 calculation module, with the GGA generalized gradient approximation and BLYP functional method. Construct an amorphous cell using the Amorphous Cell module. First, in the Geometry Optimization of the Forcite Calculation module, use the COMPASS II function to optimize the geometric structure and simulate the thermal imidization process based on the actual experimental temperature. Then, perform short-term molecular dynamics simulations under constant temperature using the NVT ensemble, followed by NVE simulations under constant energy. Finally, calculate the cohesive energy of the simulated configurations. GGA (Generalized Gradient Approximation) and BLYP (Becke-Lee-Yang-Parr) functional methods were selected as the computational parameters.

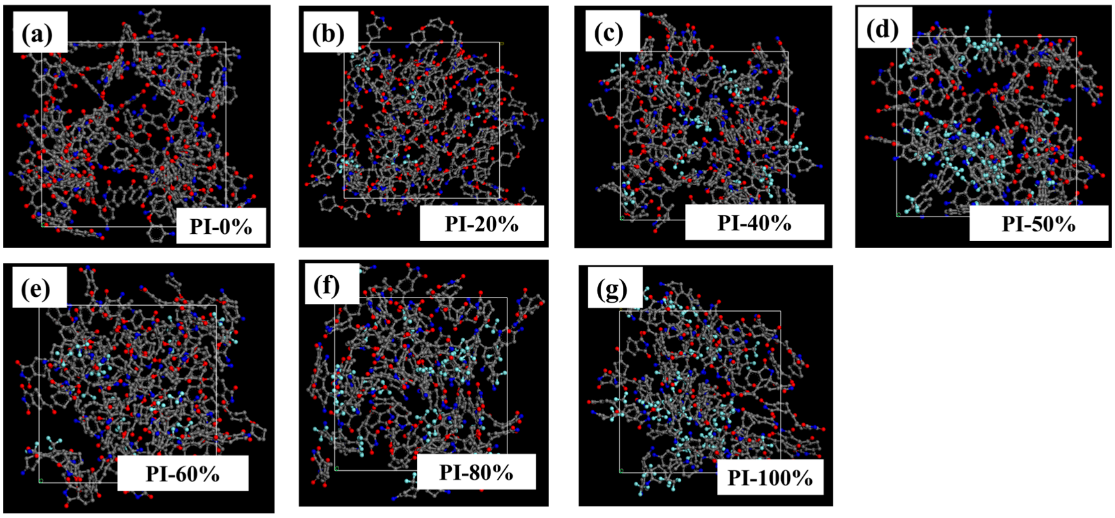

Figure S10. (a)-(f) The stereochemical structures of PI films with different copolymer obtained by DFT calculation.

The combination of first-principles simulation and machine learning for screening highly insulating polymer matrices will be a major trend in future studies of advanced materials. By rapidly and effectively selecting the optimal polymer structure, it is possible to obtain information on the microstructural evolution within the energy storage medium, investigate structure-effect relationships, facilitate the optimization of polymer microstructures, understand and predict structural evolution, and ultimately enhance the energy storage performance of polymer materials.

Table S5. The HOME and LUMO orbital energies and energy differences of different monomers were also analyzed.

	E_{HOMO} (eV)	E_{LUMO} (eV)	E_{HOMO} - E_{LUMO} (eV)
**BABP**	-5.496	-5.907	0.411
**TFMB**	-5.479	-6.874	1.395
**BTDA**	-5.396	-7.394	1.997

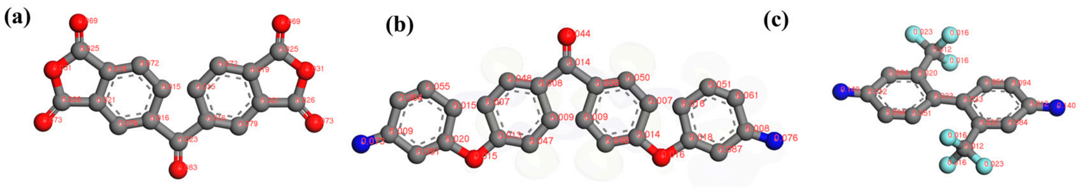

Figure S11. (a)-(c) The Fukui functions of specific atoms in the BTDA, BABP, and TFMB monomers reflect the reactivity of the local positions in the monomers.

The larger the reactivity, the greater the tendency to gain or lose electrons. The nucleophilicity and electrophilicity of a substrate can be evaluated by assessing the energy of the HOMO and LUMO respectively. The reactivity tendency can be determined by examining the energy difference between the HOMO and LUMO. A larger negative energy difference indicates a greater tendency to accept electrons.[12-13]

The cohesive energy is defined as the increase in internal energy (E) of a substance when all intermolecular forces are removed for 1 mole of the substance. It can be further defined as the cohesive energy density when calculated per unit volume.

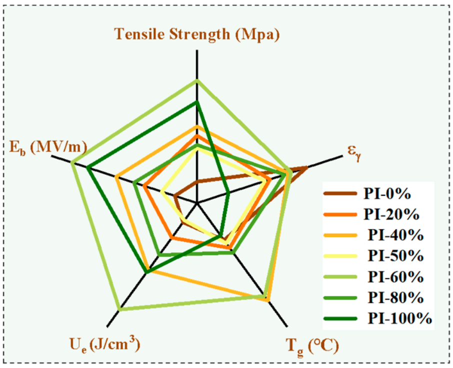

Figure S12. Radar chart of the performance of polyimides tested in different polymerization proportions

References

1. Palumbo, F.; Wen, C.; Lombardo, S.; Pazos, S.; Aguirre, F.; Eizenberg, M.; Hui, F.; Lanza, M., A Review on Dielectric Breakdown in Thin Dielectrics: Silicon Dioxide, High-k, and Layered Dielectrics. *Adv. Funct. Mater.* **2019,** *30* (18).

2. Artbauer, J., Electric strength of polymers. *J. Phys. D: Appl. Phys.* **1996,** *29* (2), 446-456.

3. Kudo, H.; Sudo, S.; Oka, T.; Hama, Y.; Oshima, A.; Washio, M.; Murakami, T., Ion-beam irradiation effects on polyimide-UV–vis and infrared spectroscopic study. *Radiat. Phys. Chem.* **2009,** *78* (12), 1067-1070.

4. Grimme, S., Semiempirical GGA-type density functional constructed with a long-range dispersion correction. *J. Comput. Chem.* **2006,** *27* (15), 1787-1799.

5. Kohn, W.; Sham, L. J., Self-Consistent Equations Including Exchange and Correlation Effects. *Physical Review* **1965,** *140* (4A), A1133-A1138.

6. Monkhorst, H. J.; Pack, J. D., Special points for Brillouin-zone integrations. *Physical Review B* **1976,** *13* (12), 5188-5192.

7. Perdew, J. P.; Chevary, J. A.; Vosko, S. H.; Jackson, K. A.; Pederson, M. R.; Singh, D. J.; Fiolhais, C., Atoms, molecules, solids, and surfaces: Applications of the generalized gradient approximation for exchange and correlation. *Physical Review B* **1992,** *46* (11), 6671-6687.

8. Wu, Z.; Guo, Q.; Liu, Y.; Zhou, H.; Zheng, H.; Lei, X.; Gong, L.; Chen, Y.; Liu, Z.; Zhang, Q., Excellent Polyimide Dielectrics Containing Conjugated ACAT for High-Temperature Polymer Film Capacitor. *Macromol. Mater. Eng.* **2021,** *306* (11).

9. Yin, C.; Dong, J.; Tan, W.; Lin, J.; Chen, D.; Zhang, Q., Strain-induced crystallization of polyimide fibers containing 2-(4-aminophenyl)-5-aminobenzimidazole moiety. *Polymer* **2015,** *75*, 178-186.

10. Qi, H.; Xie, A.; Zuo, R., Local structure engineered lead-free ferroic dielectrics for superior energy-storage capacitors: A review. *Energy Storage Materials* **2022,** *45*, 541-567.

11. Zha, J.-W.; Tian, Y.; Zheng, M.-S.; Wan, B.; Yang, X.; Chen, G., High-temperature energy storage polyimide dielectric materials: polymer multiple-structure design. *Materials Today Energy* **2023,** *31*.

12. Nørskov, J. K.; Rossmeisl, J.; Logadottir, A.; Lindqvist, L.; Kitchin, J. R.; Bligaard, T.; Jónsson, H., Origin of the Overpotential for Oxygen Reduction at a Fuel-Cell Cathode. *The Journal of Physical Chemistry B* **2004,** *108* (46), 17886-17892.

13. Yuan, L. J.; Sui, X. L.; Liu, C.; Zhuo, Y. L.; Li, Q.; Pan, H.; Wang, Z. B., Electrocatalysis Mechanism and Structure-Activity Relationship of Atomically Dispersed Metal-Nitrogen-Carbon Catalysts for Electrocatalytic Reactions. *Small Methods* **2023,** *7* (3).
